# The impact of structural optical coherence tomography changes on visual function in retinal vein occlusion

**DOI:** 10.1111/aos.14621

**Published:** 2020-09-30

**Authors:** Martin Michl, Xuhui Liu, Alexandra Kaider, Amir Sadeghipour, Bianca S. Gerendas, Ursula Schmidt‐Erfurth

**Affiliations:** ^1^ Department of Ophthalmology Vienna Reading Center and OPTIMA Study Group Medical University of Vienna Vienna Austria; ^2^ Department of Ophthalmology The First Affiliated Hospital of Zhengzhou University Zhengzhou City China; ^3^ Center for Medical Statistics Informatics and Intelligent Systems Medical University of Vienna Vienna Austria

**Keywords:** biomarker, function, morphology, optical coherence tomography, retinal vein occlusion

## Abstract

**Purpose:**

We aimed to determine the correlation between optical coherence tomography (OCT)‐ and demographic features and baseline best corrected visual acuity (BCVA) in treatment‐naïve patients with retinal vein occlusion (RVO).

**Methods:**

This was a cross‐sectional posthoc analysis of OCT images that included RVO patients from two prospective, open‐label, multicentre studies. The morphological grading was done manually, in the standardized setting of a reading centre. Main outcome measure was the estimated difference in Early Treatment Diabetic Retinopathy Study letters associated with each individual biomarker.

**Results:**

Included were 381/301 treatment‐naïve patients with BRVO/CRVO. For BRVO, statistically significant correlations with BCVA were seen for a 100 µm increase in central subfield thickness (CST; −3.1 letters), intraretinal cysts at centre point (CP; +4.1), subretinal fluid (SRF) at CP (+3.0) and hyperreflective foci (HRF) at the central B‐scan (−2.2). In CRVO, a 100 µm increase in CST was associated with a loss of −3.4 letters. In the total cohort, 100 µm increase in CST, SRF at CP and HRF at the central B‐scan correlated with a difference of −3.2,+3.2 and −2.0 letters. A 10‐year increase in age and female gender yielded a −2.0 and −2.5 letter decrease in the total cohort. Adjusted multiple *R* ^2^ for the respective group was 18.3%/26.3%/23.5%.

**Conclusions:**

Of all parameters studied, only CST and age were consistently associated with worse BCVA in treatment‐naïve RVO patients. Morphology on OCT explained only a modest part of functional loss in this patient cohort.

## Introduction

Retinal vein occlusion (RVO) is one of the most common diseases of the retinal vasculature, affecting 1–2% of the population over the age of 40 (Wong & Scott 2010). Large‐scale clinical trials have demonstrated the efficacy of anti‐vascular endothelial growth factor therapy (anti‐VEGF) in treating patients with RVO complicated by macular oedema (ME) (Brown et al. 2011; Campochiaro et al. 2011; Boyer et al. 2012; Heier et al. 2014; Ogura et al. 2014). The identification of the latter and other changes of the retinal architecture has been greatly enhanced by technological advances in optical coherence tomography (OCT), and we can now objectively assess disease progression and treatment response. Consequently, numerous studies have been targeting OCT biomarkers, such as central retinal thickness (CRT) and photoreceptor (PR) degeneration that are predictive of visual acuity after treatment and could serve as an indicator of visual recovery (Hoeh et al. 2009; Scott et al. 2011; Fujihara‐Mino et al. 2016; Liu et al. 2018). Furthermore, imaging biomarkers have been shown to play an important role in guiding ophthalmologists in tailoring treatment to suit the individual patient’s needs (Ritter et al. 2014; Waldstein et al. 2016). However, there is only scarce evidence on which morphological features are responsible for visual acuity in treatment‐naïve patients. The aim of this study is to characterize morphological features in a standardized manner and systematically evaluate the impact of these features as well as demographic parameters on best corrected visual acuity (BCVA) in a large treatment‐naïve, and well‐phenotyped cohort of RVO patients. This paper presents a comprehensive evaluation of all key OCT biomarkers in both branch and central RVO (BRVO, CRVO), that have been identified in the literature. It is hoped that this will aid the ophthalmologist in the interpretation and prioritization of structural pathologies.

## Material and methods

### Study design and population

This study, performed by the Vienna Reading Center (VRC), is a posthoc analysis of the Efficacy and Safety of Ranibizumab With or Without Laser in Comparison to Laser in Branch Retinal Vein Occlusion (Tadayoni et al. 2016) (BRIGHTER; clinicaltrials.gov identifier: NCT01599650) and Ranibizumab Intravitreal Injections in Patients With Visual Impairment Due to Macular Edema Secondary to Central Retinal Vein Occlusion (Larsen et al. 2016) (CRYSTAL; clinicaltrials.gov identifier: NCT01535261) trials, all of which were conducted in accordance with the Declaration of Helsinki and the International Conference of Harmonization of Good Clinical Practice guidelines. The study protocols of BRIGHTER and CRYSTAL were reviewed and approved by an independent ethics committee or institutional review board at each participating centre. Patients provided written informed consent before entering the clinical trials. Both studies were prospective, open‐label, multicentre studies and included only one eye per patient. Their key inclusion criterion was a BCVA letter score between 73 and 19 Early Treatment Diabetic Retinopathy Study (ETDRS) letters (approximate Snellen equivalent 20/40‐20/400) in patients 18 years of age or older.

We included all spectral‐domain (SD‐) OCT images of treatment‐naïve patients that were taken with either CIRRUS HD‐OCT (Carl Zeiss Meditec, Dublin, CA, USA; CIRRUS) or Spectralis™ OCT (Heidelberg Engineering, Heidelberg, Germany; SPECTRALIS). Images were recorded according to a predefined imaging protocol at the respective study sites by VRC‐certified personnel. In addition to imaging biomarkers, demographic factors such as age, gender and disease duration, as recorded in the core studies, were respected in our statistical analyses. Baseline BCVA letter score was defined as the dependent variable. Approval for this posthoc analysis was obtained from the Ethics Committee at the Medical University of Vienna.

### Image analysis

In our analysis, we used baseline images of BRVO and CRVO. Both CIRRUS and SPECTRALIS covered a 6 × 6 mm area with a scanning pattern of 512 A‐scans by 128/49 B‐scans. All OCT scans were graded in VRC custom software for ophthalmic image analysis, following a predefined reading protocol. The morphological grading was done manually by one experienced and trained grader (X.L.) and supervised by a senior expert (B.G.) of the VRC, who were both masked to any clinical information.

**Fig. 1:**
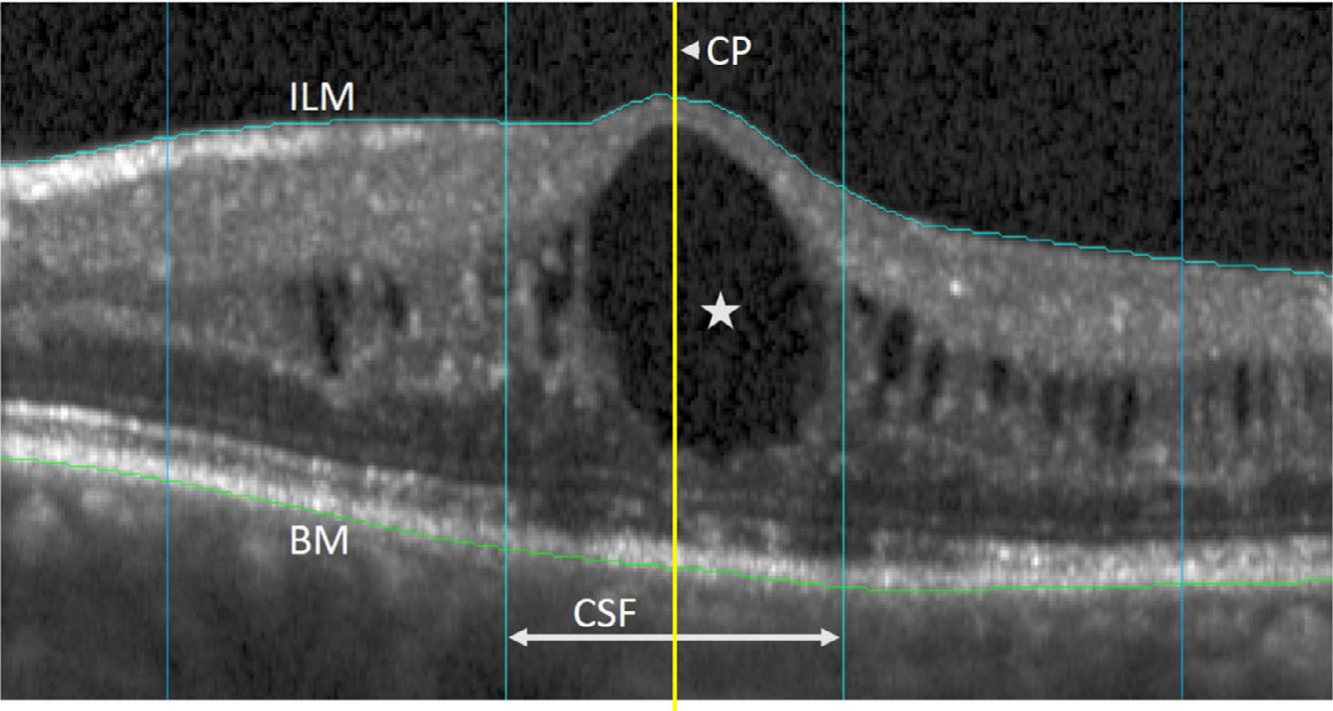
Morphological grading: corrections of misalignments of the internal limiting membrane (ILM) and Bruch’s membrane (BM) were done manually in our custom software to enable comparisons of thickness measurements between optical coherence tomography (OCT) devices. An ETDRS grid was centred on the fovea to allow for a standardized localization of OCT features at the centre point (CP, yellow line) and the central subfield (CSF). The star marks a large intraretinal cyst at the CP.

An ETDRS grid was centred on the central foveal point to allow for a standardized localization of morphological changes. Retinal thickness at the centre point (CPT) and the central subfield (CST) were measured between Bruch’s membrane and the internal limiting membrane (ILM), thus including subretinal fluid (SRF), if present. The positioning of the CP as well as corrections of alignment errors along Bruch’s membrane and the ILM were done manually in our custom software (Fig. 1). It has been shown before that this leads to good correlations of thickness measurements between devices (Simader et al. 2012).

**Fig. 2:**
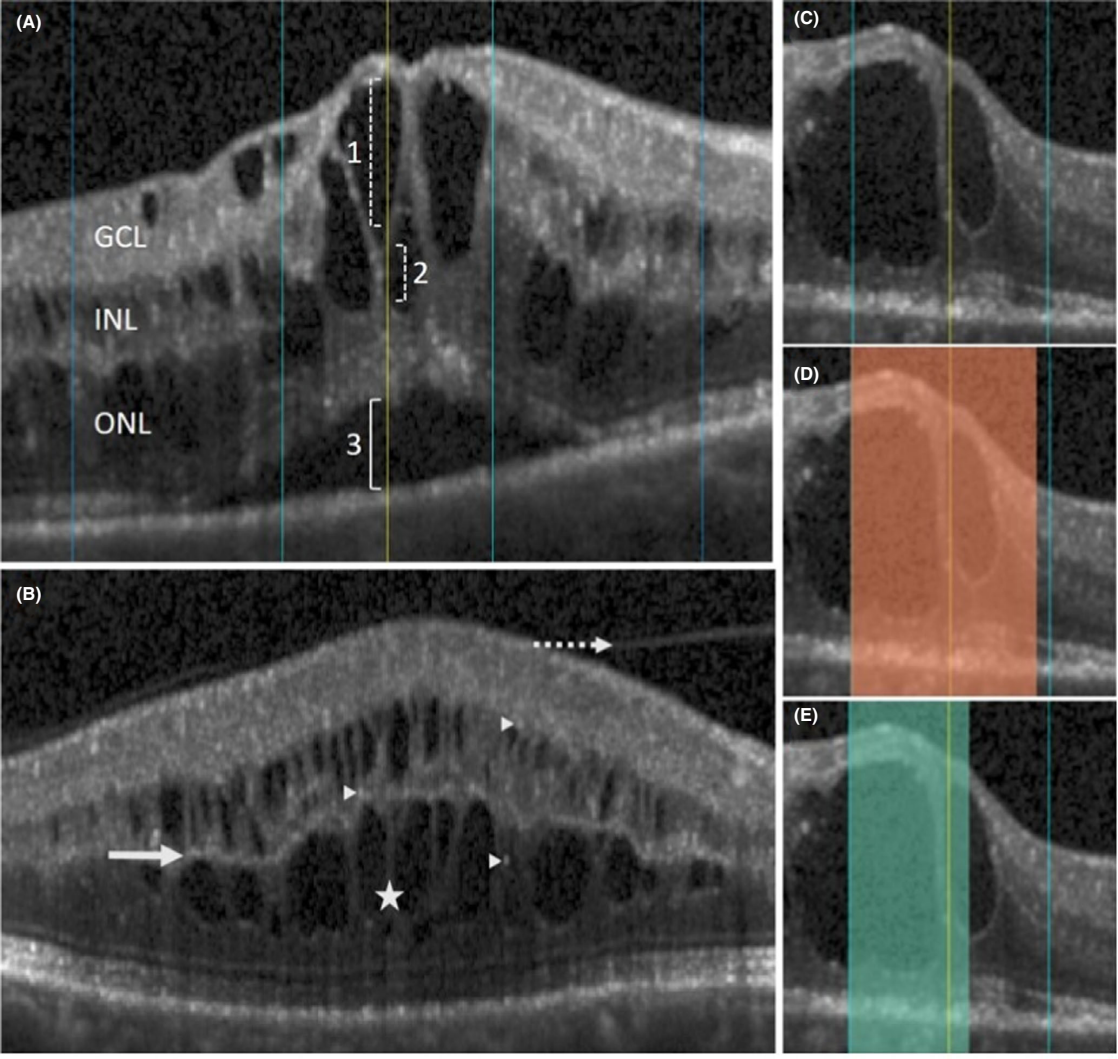
(A) There is intraretinal fluid at the centre point (CP, yellow vertical line). In case of multiple cysts at the CP, their heights (1, 2) were added together. In most cases, there were cysts seen in the ganglion cell (GCL), as well as the inner‐ and outer nuclear layer (INL, ONL). The presence of subretinal fluid (SRF) was graded within the central subfield, and its height measured at the CP (3). (B) The dotted arrow indicates detached vitreous in a patient with partial vitreous adhesion; arrow heads point at hyperreflective foci; the star shows thin septae between cysts (=processus); the bold arrow points at a prominent middle‐limiting membrane, a sign of ischaemia. (C) Photoreceptor damage within the central subfield leads to a discontinuous ellipsoid zone (disrupted area marked in D) and external limiting membrane (disrupted area marked in E).

We intended to cover all morphological features known in the literature at the time of protocol establishment. They include shape of the foveal contour (no depression/no swelling = flat retina at CSF; normal foveal depression = same aspect as healthy partner eye; small foveal depression = depression smaller than that of healthy partner eye; small foveal swelling = CPT < 450 µm; large foveal swelling = CPT ≥ 450 µm), intraretinal cystoid fluid (further referred to as cysts or IRC; cyst location/height/layer/processus = thin septae between cysts), subretinal fluid (location/height), PR integrity at the CSF (area of external limiting membrane [ELM] disruption and/or area of ellipsoid zone [EZ] disruption; in the presence of SRF, EZ disruption was only graded in case of obvious discontinuity), disorganization of retinal inner layers (DRIL) (Sun et al. 2014) at the CSF, hyperreflective foci (HRF; number on one central B‐scan), condition of the vitreomacular interface (partial or complete adhesion, traction, posterior vitreous detachment) and signs of ischaemia (prominent middle‐limiting membrane, p‐MLM (Ko et al. 2014); paracentral acute middle maculopathy, PAMM (Rahimy et al. 2014)). All area measurements (EZ‐, ELM disruption; DRIL) were done in our custom software by marking the length of the disrupted feature on each B‐scan within the CSF (Fig. 2C–E). The CSF in the centre of the ETDRS grid is one millimetre wide and thus comprises an area of 0.785 mm^2^.

### Statistical analysis

Continuous, normally distributed variables are described by the mean ± standard deviation (SD), non‐normally distributed variables by the median (quartiles). Absolute numbers and percentages are given in case of categorical variables.

To evaluate the impact of OCT biomarkers on BCVA at the treatment‐naïve stage, uni‐ and multivariable linear regression models were calculated, using the backward method for variable selection with the Akaike information criterion (AIC) as selection criteria. Within the backward selection process, variables were excluded until a local minimum of the AIC was reached. Regression models were calculated for the total study cohort, as well as separately for BRVO and CRVO patients. In a sensitivity analysis, the selection stability was assessed by computing the bootstrap inclusion frequency for each variable, obtained by repeating the backward elimination procedure in 10.000 resamples drawn with replacement from the original sample. Univariate and multiple R‐squared measures were calculated to evaluate the proportion of variation in the outcome variable explained by the considered explanatory variables.

For missing values, the method of single imputation with predictive mean matching was used to enable statistical evaluation on the total sample of all patients. Optical coherence tomography (OCT) biomarkers with more than 10% missing values were not respected in the linear regression models.

Two‐sided p‐values < 0.05 were considered as indicating statistical significance. The software SAS, version 9.4 (SAS Institute Inc., 2002–2012, Cary, NC, USA) was used for statistical calculations.

## Results

We included 682 treatment‐naïve eyes of 682 individuals with BRVO or CRVO. Of the 381/301 patients presenting with BRVO/CRVO, 233/199 underwent imaging with SPECTRALIS and 148/102 with CIRRUS. In the total cohort, mean age was 65.8 ± 11.7 years, and 292 patients (42.8%) were female. Median disease duration was 2.7 (quartiles: 0.9–9.0) months, and mean baseline ETDRS letter score was 56.1 ± 13.7 (20/80 Snellen). See Table 1 for further descriptive characteristics of the study cohort, and Table 2 for all qualitative and quantitative results of the morphological grading (Fig. 2).

**Table 1:** Descriptive characteristics of the study cohorts.

	BRVO	CRVO	Total cohort
Number of patients	381	301	682
Age, mean (SD), years	66.3 (10.5)	65.0 (13.0)	65.8 (11.7)
Gender, No. (%)			
Women	188 (49.3)	104 (34.6)	292 (42.8)
Men	193 (50.7)	197 (65.4)	390 (57.2)
Disease duration, median (IQR), months	2.9 (1.0–8.8)	2.5 (0.9–9.2)	2.7 (0.9–9.0)
Baseline BCVA, mean (SD), ETDRS letters	58.0 (12.7)	53.80 (14.5)	56.1 (13.7)

BCVA = best corrected visual acuity, BRVO = branch retinal vein occlusion, CRVO = central retinal vein occlusion, ETDRS = Early Treatment Diabetic Retinopathy Study, IQR = interquartile range, SD = standard deviation.

**Table 2:** List of manually assessed morphological biomarkers and their frequency/measurements for each cohort.

	BRVO *n* = 381	CRVO *n* = 301	Total cohort *n* = 682
Central retinal thickness			
Centre point, mean (SD), µm	554.57 (193.91)	689.07 (219.12)	624.68 (230.76)
Central subfield, mean (SD), µm	541.22 (166.15)	713.43 (243.17)	606.32 (204.78)
Foveal contour			
No depression/no swelling (%)	25 (6.6)	18 (6)	43 (6.3)
Normal foveal depression (%)	27 (7.1)	18 (6)	45 (6.6)
Small foveal depression (%)	58 (15.2)	49 (16.3)	107 (15.7)
Small foveal swelling (%)	137 (35.9)	62 (20.6)	199 (29.2)
Large foveal swelling (%)	125 (32.8)	148 (49.1)	273 (40)
Not gradable (%)	9 (2.4)	6 (2)	15 (2.2)
Intraretinal fluid			
Presence (%)	376 (98.7)	297 (98.7)	673 (98.7)
1 cyst at CP (%)	282 (74)	238 (79.1)	520 (76.3)
2 or 3 cysts at CP (%)	16 (4.2)	14 (4.7)	20 (2.9)
Cyst height at CP, median (IQR), µm*	283 (204–393)	430 (230–441)	304 (211–414)
Affection of INL + ONL+GCL (%)^†^	314 (82.4)	282 (93.7)	596 (87.4)
Small cysts, ≤200 µm (%)	69 (18.1)	47 (15.6)	116 (17)
Medium cysts, 201–400 µm (%)	145 (38.1)	114 (37.9)	259 (38)
Large cysts, >400 µm (%)	68 (17.9)	77 (25.6)	145 (21.3)
Presence of cystoid processus (%)	317 (83.2)	266 (88.4)	583 (85.5)
Subretinal fluid			
Presence (%)	171 (44.9)	198 (65.8)	369 (54.1)
Location at CP (%)	143 (37.5)	184 (61.1)	327 (47.9)
Location at CSF but not CP (%)	12 (3.1)	3 (1)	15 (2.2)
Height at CP, median (IQR), µm	157 (105–251)	178 (112–284)	174 (110–271)
Photoreceptors			
Disrupted ELM in CSF (%)	316 (83)	178 (59.1)	494 (72.4)
Area of ELM disruption in CSF, median (IQR; % of CSF), mm^2^	0.606 (0.433–0.732; 72.3)	0.711 (0.520–0.789; 79.3)	0.649 (0.454–0.759; 74.9)
Disrupted EZ in CSF (%)	318 (83.5)	181 (60.1)	499 (73.2)
Area of EZ disruption in CSF, median (IQR; % of CSF), mm^2^	0.667 (0.453–0.786; 75.1)	0.758 (0.525–0.788; 80.9)	0.700 (0.477–0.787; 77.3)
ELM not gradable (%)	60 (15.8)	111 (36.9)	171 (25.1)
EZ not gradable (%)	58 (15.2)	109 (36.2)	167 (24.5)
DRIL			
Presence (%)	344 (90.3)	229 (76.1)	573 (84)
Area of disruption in CSF, median (IQR; % of CSF), mm^2^	0.640 (0.458–0.780; 76)	0.788 (0.759–0.798; 92.4)	0.744 (0.549–0.788; 82.6)
Hyperreflective foci			
Presence on central B‐scan (%)	372 (97.6)	269 (89.4)	641 (94)
Number on central B‐scan, mean (SD)	32.4 (19)	39.6 (17)	35.5 (18)
Vitreomacular interface			
Total adhesion (%)	88 (23.1)	67 (22.3)	155 (22.7)
Partial adhesion (%)	54 (14.2)	38 (12.6)	92 (13.5)
PVD (%)	33 (8.6)	22 (7.3)	55 (8.1)
Traction (%)	9 (2.4)	8 (2.7)	17 (2.5)
Not gradable (%)^‡^	195 (51.2)	166 (55.1)	361 (52.9)
Missing (%)^‡^	2 (0.5)	0	2 (0.3)
Ischaemia			
p‐MLM (%)	165 (43.3)	179 (59.5)	344 (50.4)
PAMM (%)	2 (0.2)	2 (0.7)	4 (0.6)

µm = microns, BRVO = branch retinal vein occlusion, CP = centre point, CRVO = central retinal vein occlusion, CSF = central subfield, DRIL = disorganization of retinal inner layers, ELM = external limiting membrane, EZ = ellipsoid zone, GCL = ganglion cell layer, INL = inner nuclear layer, IQR = interquartile range, mm^2^ = square millimetre, ONL = outer nuclear layer, PAMM = paracentral acute middle maculopathy, p‐MLM = prominent middle‐limiting membrane, PVD = posterior vitreous detachment, SD = standard deviation.

* Summative if > 1 cyst.

^†^ Cases that showed cysts in the INL, ONL and GCL.

^‡^ Not gradable = vitreous is visible but image quality is insufficient for assessment of vitreomacular interface.

^‡^ Missing = vitreous and vitreomacular interface are not visible.

### Regression analysis

The impact of the individual OCT‐ and demographic features on BCVA, as calculated in the univariate analysis, are shown in Table 3. The variation in baseline VA that can be explained by all morphologic and demographic features was 18.3% for BRVO, 26.3% for CRVO and 23.5% for the total cohort (Table 4).

**Table 3:** Univariate correlations between the individual variables featured in each model and best corrected visual acuity.

Variable (unit)	BRVO (*n* = 381)				CRVO (*n* = 301)				Total cohort (*n* = 682)			
	Regr. Coeff.	SE	p Value	*R* ^2^	Regr. Coeff.	SE	p Value	*R* ^2^	Regr. Coeff.	SE	p Value	*R* ^2^
CRT at CSF; (100 µm)	**−2.4**	**0.4**	**<0.0001**	**9.8**	**−3.1**	**0.3**	**<0.0001**	**22.6**	**−2.8**	**0.2**	**<0.0001**	**17.9**
CRT at CP; (100 µm)	**−1.8**	**0.3**	**<0.0001**	**7.9**	**−2.6**	**0.3**	**<0.0001**	**19.6**	**−2.3**	**0.2**	**<0.0001**	**15.4**
IRC at CP: presence versus absence	+0.2	1.5	0.87	0.01	**−5.8**	**2**	**0.004**	**2.7**	**−2.5**	**1.2**	**0.043**	**0.6**
IRC height at CP; (100 µm)	**−1.9**	**0.5**	**0.0003**	**3.5**	**−1.8**	**0.6**	**0.0001**	**5.9**	**−2**	**0.4**	**<0.0001**	**4.5**
IRC in GCL, presence versus absence	−0.7	1.8	0.696	0.04	**−10**	**3.5**	**0.004**	**2.7**	**−3.9**	**1.6**	**0.016**	**0.9**
Cystoid processus at CSF (presence versus absence)	−1.3	1.8	0.491	0.1	+0.03	2.9	0.99	0	−1.4	1.6	0.374	0.1
Foveal contour:			**0.004**	**4**			**0.007**	**4.7**			**<0.0001**	**4.5**
Normal foveal contour	+5	3.3			+4.8	4.5			+4.9	2.7		
Small foveal depression	+7.8	3			+3	3.9			+5.7	2.4		
Small foveal swelling	+4.9	2.7			−0.7	3.8			+3	2.3		
Large foveal swelling	+1	2.7			−4	3.5			−1.6	2.2		
SRF (at CP versus no SRF/SRF off CP)	−1	1.3	0.45	0.2	−3.6	1.9	0.052	1.3	**−3.1**	**1**	**0.004**	**1.3**
HRF (30 foci)	**−4.1**	**1**	**<0.0001**	**4.2**	**−4.8**	**1.5**	**0.001**	**3.4**	**−4.9**	**0.8**	**<0.0001**	**4.8**
Vitreomacular interface:			0.098	1.7			0.508	0.8			0.079	1.0
Vitreous adhesion	+2.1	1.6			+3.1	2.1			+2.7	1.3		
Vitreous adhesion, partial	+2.4	1.9			+0.7	2.6			+1.9	1.6		
Posterior vitreous detachment/vitreous traction	−3.1	2.1			+0.2	2.9			−1.5	1.7		
Age (10 years)	**−2.0**	**0.6**	**0.001**	**2.7**	**−1.7**	**0.6**	**0.009**	**2.3**	**−0.2**	**0.4**	**0.0001**	**2.1**
Female gender	**−2.8**	**1.3**	**0.035**	**1.2**	−1.9	1.8	0.276	0.4	−1.7	1.1	0.103	0.4
Disease duration (log2 transformed)*	+0.3	0.3	0.259	0.3	−0.3	0.4	0.456	0.2	+0.1	0.2	0.707	0.02

Statistically significant results are shown in bold.

BRVO = branch retinal vein occlusion, CP = centre point, CRT = central retinal thickness, CRVO = central retinal vein occlusion, CSF = central subfield, GCL = ganglion cell layer, HRF = hyperreflective foci, IRC = intraretinal cysts, *R* ^2^ = R‐squared, Regr. Coeff. = regression coefficient, SE = standard error, SRF = subretinal fluid, μm = microns.

* Difference in letters per doubling of disease duration.

**Table 4:** Correlations between the variables featured in each final multivariable regression model and best corrected visual acuity.

Variable (unit)	BRVO (*n* = 381)			CRVO (*n* = 301)			Total cohort (*n* = 682)		
	Regression coefficient	Standard error	p Value	Regression coefficient	Standard error	p Value	Regression coefficient	Standard error	p Value
CRT at CSF; (100 µm)	**−3.1**	**0.5**	**<0.0001**	**−3.4**	**0.4**	**<0.0001**	**−3.4**	**0.3**	**<0.0001**
IRC at CP: presence versus absence*	**+4.1**	**1.7**	**0.017**	^§^	^§^	^§^	^§^	^§^	^§^
Cystoid processus at CSF (presence versus absence)	^§^	^§^	^§^	+4.3	2.6	0.1	^§^	^§^	^§^
Foveal contour^†^			0.1						**0.03**
Normal foveal contour	+3.2	3.4		^§^	^§^	^§^	**−**2.0	2.5	
Small foveal depression^‡^	+7.3	2.8		^§^	^§^	^§^	+3.5	2.2	
Small foveal swelling	+4.8	2.5		^§^	^§^	^§^	+1.5	2.0	
Large foveal swelling	+5.1	2.6		^§^	^§^	^§^	+3.3	2.0	
SRF (at CP versus no SRF/SRF off CP)	**+3.0**	**1.4**	**0.029**	+3.0	1.9	0.109	**+3.2**	**1.1**	**0.003**
HRF (30 foci)	**−2.2**	**1.0**	**0.027**	**−**1.9	1.3	0.159	**−2.0**	**0.8**	**0.014**
Age (10 years)	**−2.3**	**0.6**	**<0.0001**	**−1.6**	**0.6**	**0.004**	**−2.0**	**0.4**	**<0.0001**
Female gender	**−3.4**	**1.2**	**0.005**	^§^	^§^	^§^	**−2.5**	**0.9**	**0.008**
Disease duration (log2 transformed) ^‡^	^§^	^§^	^§^	**−**0.7	0.3	0.057	^§^	^§^	^§^
Adjusted multiple *R*‐squared	18.3%			26.3%			23.5%		

Regression coefficients indicating the difference in ETDRS letters; adjusted multiple R‐squared indicating the overall performance of the individual model. Statistically significant results are shown in bold.

BRVO = branch retinal vein occlusion, CP = centre point, CRT = central retinal thickness, CRVO = central retinal vein occlusion, CSF = central subfield, HRF = hyperreflective foci, IRC = intraretinal cysts, SRF = subretinal fluid, μm = microns.

* See ‘Discussion’ for an explanation of contrasting effects between ‘CRT at CSF’ and ‘presence of IRC at CP’

^†^ When compared to ‘no depression/no swelling’.

^‡^ Statistically significant when compared to other changes of foveal contour.

^‡^ difference in letters per doubling of disease duration.

^§^ Variable was excluded in backward selection process and is thus not featured in the final regression model of the respective cohort.

In BRVO, an increase in CRT of 100 µm at the CSF was associated with a −3.1 letter drop. The presence of IRC at the CP correlated with a +4.1 letter gain. A small foveal depression, when compared to a normal foveal contour, and the presence of SRF at the CP led to a +7.3 and + 3.0 letter gain, respectively. The presence of at least 30 HRF on one central B‐scan correlated with a −2.2 letter loss. Female sex and a 10‐year increase in patients’ age were associated with a differences of −3.4 and −2.3 letters, respectively.

In the CRVO model, an increase in CRT of 100 µm at the CSF was correlated with a drop of −3.4 letters. A 10‐year increase in patients’ age yielded a difference of −1.6 letters.

In the total study cohort, an increase in CRT of 100 µm at the CSF correlated with a −3.4 letter loss. The presence of SRF at the CP was associated with a gain of +3.2 letters. The presence of at least 30 HRF on one central B‐scan correlated with a −2.0 letter loss. Female sex and a 10‐year increase in patients’ age yielded a reduction of −2.5 and −2.0 letters, respectively.

Using the bootstrap resampling method as a sensitivity analysis, the selection stability of the independent variables was assessed. The higher the selection percentage value of a biomarker, the more relevance can be attributed to this individual marker. Biomarkers that were statistically significant in the multivariable linear regression models showed a high inclusion frequency: in BRVO, selection percentages were 90% or higher, except for foveal contour (69%)*;* in CRVO, selection percentages were 100% for CSF, and 85% for age; the total cohort showed selection percentages above 84%, and 100% for CSF.

## Discussion

In the study described herein, we investigated the effect of morphological features as well as demographic parameters on BCVA in treatment‐naïve patients with BRVO and CRVO. Our rigorous correlation is the first to include an extensive set of OCT biomarkers that were evaluated in a homogenous large patient cohort. Further strengths of our analysis comprise the data acquisition according to a strict protocol, the similar inclusion criteria in the core trials, as well as the highly standardized setting of a professional reading centre, in which the image analysis has been carried out.

From all OCT biomarkers that were included in our analysis, an increase in CRT was associated with a loss in baseline BCVA of up to −3.4 ETDRS letters and was related to an accumulation of intra‐ (98%) and subretinal fluid (54%). A similar, albeit less pronounced effect on visual function has been reported in the SCORE study, in which every 100 µm increase in CPT led to a reduction of −1.7 (CRVO) and −1.9 letters (BRVO) (Scott et al. 2009). In terms of its spatial distribution within the neurosensory retina, IRC equally affected the ganglion cell, inner nuclear and outer nuclear layer in 87% of all eyes, indicating a rapid and aggressive expansion of fluid in RVO. This is supported by a relatively high prevalence of SRF, a feature that has been considered a sign of progression in the context of ELM disruption and unhindered migration of fluid into the subretinal space (Gass 1999; Tsujikawa et al. 2010). Even though an ELM disruption was more frequently seen in BRVO than CRVO (83% and 59%, respectively), SRF was more often detected in the latter (45% and 66%, respectively), suggesting that additional factors might play a role in the formation of SRF. The ‘protective’ effect of SRF on vision was an unexpected finding in our study and hints at a more complex aetiology and role of SRF in RVO (Hoeh et al. 2010; Liu et al. 2015; Philip et al. 2016). These findings establish RVO as an acute condition that is, above all, driven by fluid invasion. This is in contrast to findings of other comprehensive correlations of morphology and function in diseases characterized by a more degenerative nature: in nAMD, CRT was not significantly correlated with vision (Gerendas et al. 2018), whereas in DME, CRT was merely part of a larger set of biomarkers that all had a statistically significant effect on vision (Gerendas et al. 2017).

With anti‐VEGF medication as the mainstay of treatment in RVO, fluid might further represent the bridging element between the pronounced structural alteration and visual recovery following fluid resolution. This is supported by numerous studies that have identified baseline CRT as a predictive factor for BCVA after anti‐VEGF therapy (Ach et al. 2010; Hoeh et al. 2010; Fujihara‐Mino et al. 2016). It thus comes as a surprise that the parameters in our models account only for a relatively small amount of variability (R‐squared) in baseline visual acuity. Despite the inclusion of numerous OCT features and also demographic parameters in our models, other retinal abnormalities, like those seen in OCT angiography might play a more significant role in this vascular disease (Tsai et al. 2018).

Although the increase in CRT is highly dependent upon the presence of IRC, these features exhibited contrasting effects on BCVA letter score in the BRVO model (see Table 3 ‘IRC at CP’). It is essential to be aware that in a multivariable linear regression model like ours, the individual parameter estimate applies only when all other parameters are kept constant. In other words, the gain in letters associated with the presence of IRC at the CP, as calculated in our model, implies constant CRT values. In light of IRC‐presence being highly correlated with CRT, this particular finding cannot be deemed clinically important.

Despite the growing body of evidence demonstrating PR integrity as an important predictor of VA after treatment (Ota et al. 2007; Yamaike et al. 2008; Sakamoto et al. 2009; Shin et al. 2011; Wolf‐Schnurrbusch et al. 2011; Kang et al. 2013; Kim et al. 2018), EZ and ELM disruption were not featured in our multiple regression analyses. This might partly be due to the limited assessability of PRs, their preserved oxygenation via the choroid and, possibly, a ‘protective’ effect of SRF. The latter phenomenon has previously been described for nAMD (Jaffe et al. 2013; Schmidt‐Erfurth et al. 2015; Klimscha et al. 2017). Another relatively novel OCT feature that has recently received increased attention is DRIL. However, we believe that the key factor preventing DRIL from being a clinically relevant biomarker is its imprecise definition: previously characterized as the lack of distinguishable boundaries between the inner retinal layers (Sun et al. 2014), it can in fact be caused by many different features, such as IRC that alter retinal boundaries, an increase or decrease of optical intensity, HRF or a generalized blurring of layers that presents as a homogenous mass (Abdulaal et al. 2015; Chen et al. 2018). All of these features might have different implications on visual function or treatment response and complicate the standardized assessment of such a ‘nonfeature’ (Schmidt‐Erfurth & Michl 2019).

With ischaemia being a main driver of negative sequelae in RVO, numerous studies have stressed the importance of early treatment for a greater structural and functional recovery (Pikkel et al. 2013; Ogura et al. 2014; Larsen et al. 2016; Qin et al. 2017; Tadayoni et al. 2017). The higher frequency of a p‐MLM sign, as well as the marginally significant negative impact of disease duration on vision highlight the relevant ischemic drive in CRVO (Campochiaro et al. 2009; Funk et al. 2009). P‐MLM has further been described as an indicator of acute retinal ischemic damage, appearing and disappearing within one month of disease onset (Ko et al. 2014). This was clearly not the case in our study cohort, where the median disease duration of 2.7 months in both patients with and without a p‐MLM indicated less of a time dependence. Other studies reported on the negative impact of duration of ME on visual outcomes (Yeh et al. 2012; Chatziralli et al. 2018). Surprisingly, no such relation was seen in our analysis. In terms of anti‐VEGF treatment, patients in the BRIGHTER and CRYSTAL studies were only excluded if they had received such treatment within three months prior to baseline. We believe that this could have led to the inclusion of patients who had received injections at an even earlier timepoint and thus exhibited different levels of disease activity and visual impairment at study baseline.

Our results further corroborated findings from previous studies that have reported an adverse effect of advanced age (Hoeh et al. 2009; Daien et al. 2012; Chatziralli et al. 2016; Wang et al. 2016) and the presence of HRF (Kang et al. 2014; Chatziralli et al. 2016; Mo et al. 2017) on retinal function in patients with RVO. The negative association of female gender and baseline BCVA may suggest the presence of structural qualities or processes in the retina that are gender‐specific (Poplin et al. 2018).

Relevant differences in feature frequencies between the two OCT modalities were only seen for a p‐MLM sign. Its detection by SPECTRALIS exceeded that of CIRRUS by 11% and is believed to be due to the higher signal to noise ratio of the former OCT device and the often subtle presentation of the feature. All other differences were below 9%. Features with more than 10% missing data were not included in our models and comprise SRF height, area of ELM and EZ disruption, area of DRIL and signs of ischaemia. The main reasons for reduced assessability of a feature were low image quality or the coinciding presence of other pathologies, such as cystoid changes. Another limitation of our study is its retrospective design and associated drawbacks such as selection bias and lack of data.

In conclusion, our large‐scale analysis is the first to include a plethora of OCT biomarkers in RVO that were evaluated in a rigorous manner and highly standardized setting. Structurally, an increase in CRT and the presence of subretinal fluid as well as HRF correspond markedly with visual function. This differs from other retinal diseases such as nAMD or DME, where other, more complex imaging biomarkers seem to have a more distinct impact on vision. Considering findings from studies that have examined the predictive value of morphological alterations over time, reviewing OCT images for the presence of fluid is therefore critical in the monitoring and treatment of RVO patients.

However, confronted with an increasing amount of image data in clinical practice, a thorough OCT assessment by a clinician that accounts for absolute volume of retinal fluid has become largely unrealistic. Our goal should thus be the implementation of a fully automated approach that not only detects fluid with high accuracy and reliability, but also enables the assessment of its change over time (Vogl et al. 2017; Schlegl et al. 2018).

High‐resolution OCT imaging holds substantial amounts of information whose interpretation exceeds human capabilities. This might also be reflected by our models’ performances (R‐squared) that suggest the contribution of other, potentially structural elements, to the visual decline in RVO. One way to overcome this shortfall is the application of deep learning‐based methods that allow an automated feature extraction by the use of all pixel information, and thus a better understanding of the role of morphological changes in retinal disease.
